# [18-C-6H_3_O^+^]: an in-situ generated macrocyclic complex and an efficient, novel catalyst for synthesis of pyrano[2,3-*c*]pyrazole derivatives

**DOI:** 10.1038/s41598-020-70665-z

**Published:** 2020-08-31

**Authors:** Manisha Mishra, K. J. Jomon, V. R. Sriram Krishnan, Aatika Nizam

**Affiliations:** Department of Chemistry, CHRIST (Deemed to be University), Hosur Road, Bangalore, 560029 India

**Keywords:** Catalysis, Synthetic chemistry methodology

## Abstract

Synthesis of small aromatic heterocycles is of greater importance in the organic chemistry due to their vibrant applications in pharmaceuticals, agrochemicals and veterinary products. Pyranopyrazoles are one such class of heterocycles associated with numerous applications. Hence herein we report a multicomponent crown ether catalyzed, ultrasound irradiated methodology to make different functionalized pyranopyrazoles in a single step. This technique involves the in-situ generation of [18-C-6H_3_O^+^][OH^−^] complex, which in turn activates the aromatic aldehyde and aids in the facile nucleophilic addition.

## Introduction

**Figure 1:**
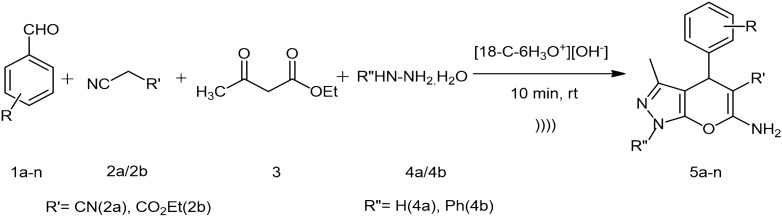
Schematic representation for the synthesis of pyrano[2,3-*c*]pyrazoles.

**Figure 2:**
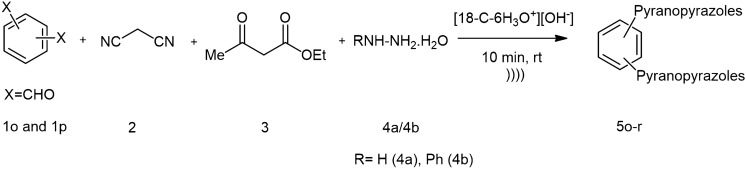
Synthesis of bis-pyrano[2,3-*c*]pyrazoles.

The physical properties of pyranopyrazoles (pypys) have rendered them as highly efficient pharmacophores, for the treatment of cancer, diabetic, pyretic, HIV, etc^1^. The existing synthetic methods for the generation of pypys requires long reaction time, tedious purification and tough conditions^2–11^, therefore the development of a straightforward method is sought for. Among macrocycles, crown ethers are of great value in synthetic chemistry field as phase transfer catalyst. The ionic-liquid type behaviour of crown ethers by forming complexes with metals and molecular cations opened a new door to the catalysis world. The importance of crown ether cation complexed ionic-liquids (CECILs) was known from the past but the real application in organic synthesis was explored by Jing et al. in 2011^12^. Later in 2017 Abaszadeh and Mohammad synthesized 1,4-dihydropyridines and tetrahydro-4*H*-chromenes using some CECILs^13^. In continuation of our work on the development of new and simple catalyst for the synthesis of bioactive compounds^14–17^, also taking inspiration from K Nikoofar’s work on the preparation of Spiro[indoline-3,2′-quinoline] substrates using [DB-18-C-6K^+^][OH^−^]nIL^18^ and noting the fact that very little is explored in this, herein we report the in-situ generation of crown ether hydronium ion complex ([18-C-6H_3_O^+^][OH^−^]) and it’s catalytic activity in four-component synthesis of pypys under sonication (Fig. 1). The key to the success of this method is the synthesis of bis-pypys, which was also established using the developed catalytic system (Fig. 2).

## Results and discussion

**Figure 3:**
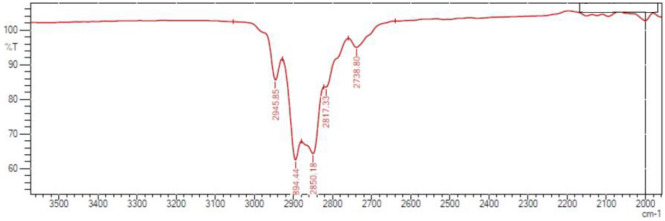
FTIR spectrum of [18-C-6H_3_O^+^][OH^−^] high frequency region.

We began our investigation by performing a model reaction using benzaldehyde (**1a**), **2a**, **3** and **4a** as substrates, and 2 mol% of 18-crown-[6]-ether/H_2_O in methanol under ultrasound irradiation for 10 min. We were encouraged by the formation of **5a** in 52% yield. Further sonication did not show any changes in the product yield. In order to confirm the influence of the catalytic system few control experiments were carried out. First, the reaction was conducted without crown ether/H_2_O, trace amount of product was formed after 25 min. About 35% of the product was obtained when the reaction was carried out only with water, and 20% yield was obtained when only crown ether was used (Table 1, entries 1–4). This observation led us to suggest the in-situ generation of [18-C-6H_3_O^+^][OH^−^] complex and its catalytic activity towards this transformation. The literature provided strong support to our suggestion^19–23^. Normally hydronium ions have a very short lifetime, ~ 10^−12^ s^24^ but by the addition of crown ether, the short-living hydronium ions get stabilized by forming a complex with it and stays indefinitely long at room temperature^19,24^. To confirm the formation of [18-C-6H_3_O^+^][OH^−^] complex we recovered the catalyst from the filtrate and subjected for FTIR analysis. The strong band at the 2,850 cm^−1^ showed the presence of H_3_O^+^ in the crown ether. This was compared with the results published by Robert Chenevert and Andre Rodrique to establish the presence of H_3_O^+^ ion in the 18-crown-6-H_3_O^+^-BF_4_ complex^24^ and was found to be in good agreement (Fig. 3).

**Table 1:** Catalyst loading and solvent screening for pyrano[2,3-*c*]pyrazole synthesis.

Entry	Solvent	Catalyst (mol %)	Time (min)	Reaction condition	Yield (%)
1	MeOH	2	10	rt/))))	52
2	MeOH	–	25	rt/))))	Trace
3	MeOH	Water only	10	rt/))))	35
4	MeOH	18-C-6 only	10	rt/))))	20
5	CH_3_CN	2	10	rt/))))	52
6	CH_2_Cl_2_	2	10	rt/))))	55
7	Toluene	2	10	rt/))))	60
8	DMSO	2	10	rt/))))	66
9	DMF	2	10	rt/))))	63
10	H_2_O	2	10	rt/))))	71
11	H_2_O	4	10	rt/))))	80
12	H_2_O	6	10	rt/))))	83
13	H_2_O	8	10	rt/))))	88
14	**H**_**2**_**O**	**10**	**10**	**rt/))))**	92*
15	H_2_O	12	10	rt/))))	90
16	H_2_O	14	10	rt/))))	90
17	H_2_O	10	40	Reflux	50

*Reaction condition-Benzaldehyde (2 mmol), malononitrile (2 mmol), ethyl acetoacetate (2 mmol), hydrazine hydrate (2 mmol) and 18-Crown-[6]-ether (10 mol%) in 10 ml of water at RT under ultrasonication.

A series of reactions were carried out in order to find a suitable solvent for this reaction and concluded that water itself is the best medium, which made our methodology more eco-friendly and greener (Table 1, entries 5–10). After choosing the catalyst and solvent the reaction progress was checked with different quantities of 18-crown-[6]-ether and found that the reaction profile improved considerably from 2 to 10 mol%, but further increase did not make any effect on the yield (Table 1, entries 10–16). Finally, it was concluded that 10 mol% of the catalyst is required on 2 mmol scale reaction. To confirm the effect of ultrasound waves on the reaction kinetics one reaction under reflux condition was conducted, desired product formation was observed after 40 min by checking TLC (Table 1, entry 17).

**Figure 4:**
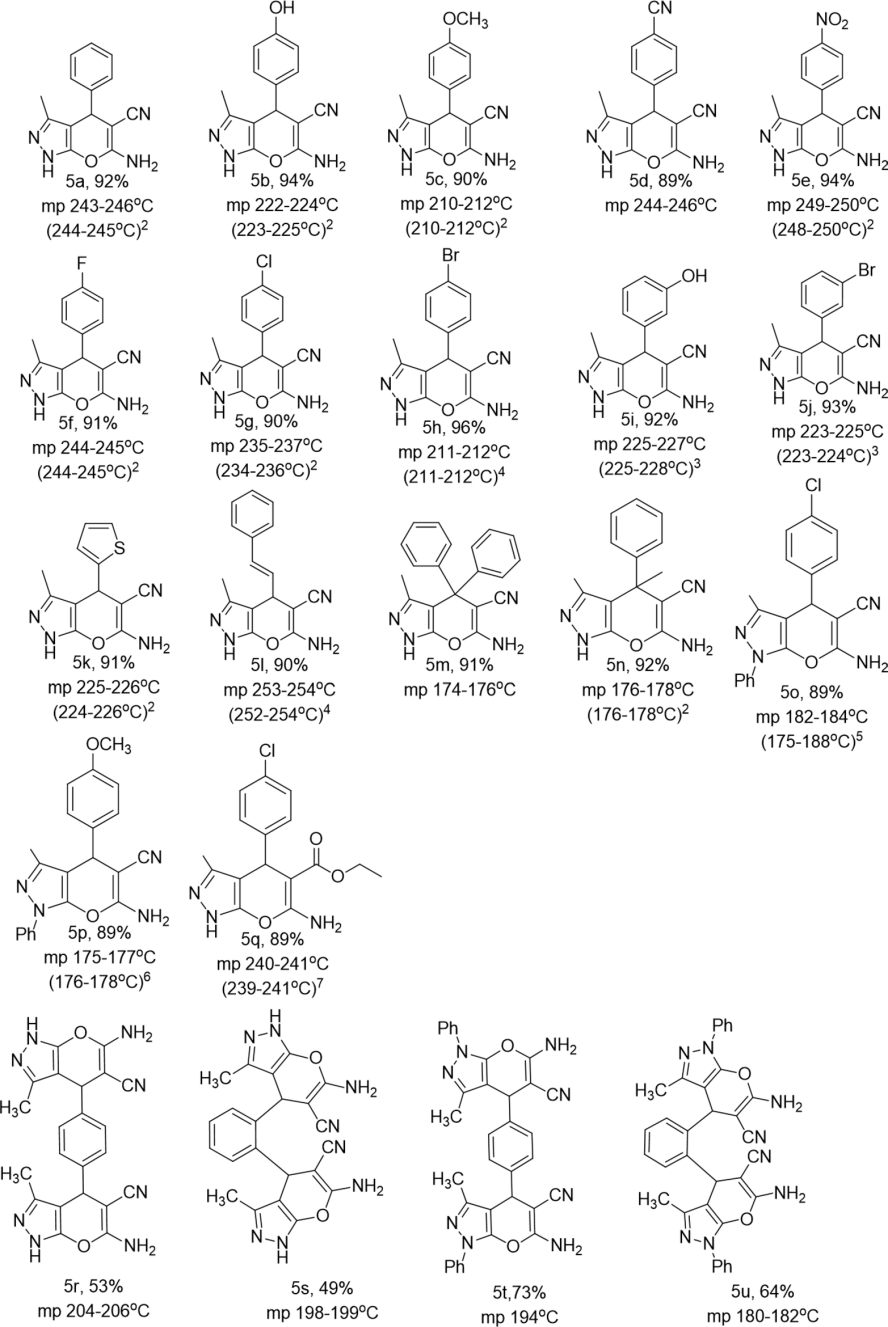
Substrate scope for pyrano[2,3-*c*]pyrazoles synthesis catalyzed by [18-C-6H_3_O^+^][OH^−^] under ultrasonic irradiation.

Intrigued by the formation of desired product **5a**, we examined the generality of the reaction with various substrates using the optimized conditions. Aldehydes bearing electron-donating groups and electron-withdrawing groups (**1b–1j**) were examined and gratifyingly all were well tolerated to furnish good yield of products (**5b–5j**) (Fig. 4). Moreover, heteroaryl aldehyde (**1k**) and cinnamaldehyde (**1l**) also provided the corresponding pypys (**5k** and **5l** respectively) in good yield.

Furthermore, employing ketones (**1m, 1n**) in place of aldehyde resulted in the formation of a quaternary carbon center at the pyran ring in the product (**5m, 5n**), which made our method much more interesting. After the successful demonstration of the feasibility of aromatic aldehydes and ketones for this strategy. We employed phenyl hydrazine (**4b**) and ethyl cyanoacetate (**2b**) in the reaction (Fig. 1). The results were satisfying giving good yield of the products (**5o–5q**). The formation of products (**5o–5q**) were confirmed by comparing their melting points with reported melting points^5–7^. Next, we were interested in the synthesis of *bis*-pypys with the established conditions by using *bis*-aldehyde functionalities (Fig. 2). For that terephthaldehyde (**1o**) and phthalaldehyde (**1p**) were chosen and furnished the respective *bis*-pypys (**5r, 5s**) in moderate yield. The study was further extended to synthesize *N*-arylated pypys using phenyl hydrazine (**4b**) instead of hydrazine hydrate (**4a**) under the same conditions and to our delight, the formation of **5t** was observed in 73% and **5u** in 64% yield.

**Figure 5:**
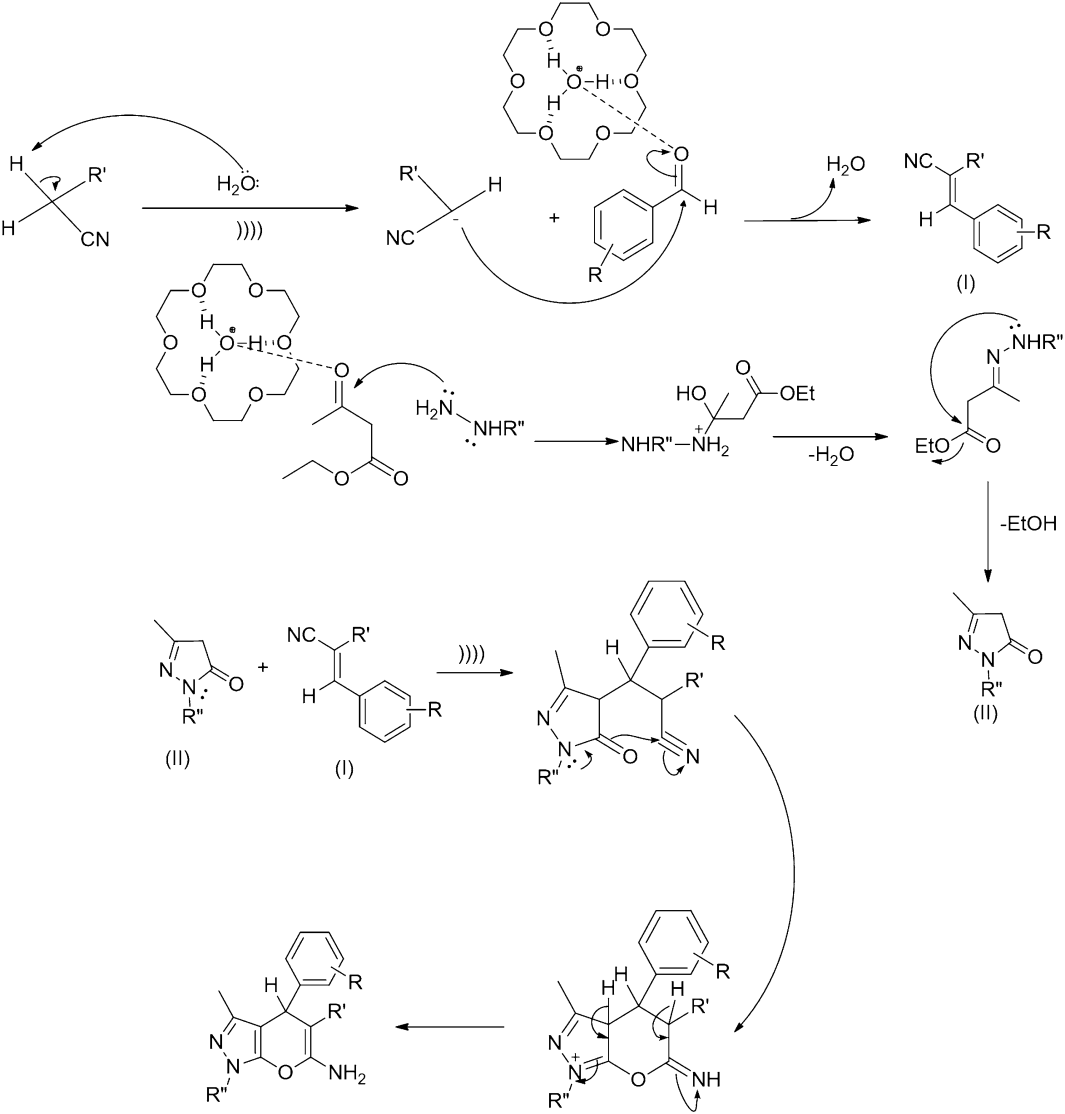
Plausible mechanism for the formation of pyrano[2,3-*c*]pyrazoles catalyzed by [18-C-6H_3_O^+^][OH^−^].

Based on the observations and literature reports, we propose a pathway, that first involves the C-H activation of malononitrile by lewis basic water molecule and activation of aromatic aldehyde by the [18-C-6H_3_O^+^][OH^−^] complex, which resulted in the formation of Knoevenagel adduct (**I)**. Further [18-C-6H_3_O^+^][OH^−^] activates the ethyl acetoacetate and speeds up the formation of pyrazolone (**II)**. Michael addition of pyrazolone (**II**) with Knoevenagel adduct (**I**) and finally cyclization and tautomerization gives the final product (Fig. 5).

In order to elaborate the synthetic application of our strategy, a 10g-scale synthesis of **5a** was demonstrated and delightfully the yield was reproduced which clearly indicates that there is a substantial potential for industrial application. All the products were confirmed by comparing their physical and spectroscopic data with reported data.

## Conclusion

We have successfully developed a simple, expeditious and green method for the synthesis of various pyrano[2,3-*c*]pyrazoles and *bis*-pyrano[2,3-*c*]pyrazoles by using 18-Crown-[6]-ether under ultrasonication condition in the aqueous medium. The catalyst generated within the reaction medium boosted up the reaction rate and yield. The crown ether hydronium ion complex are known but the catalytic activity of these complex for the synthesis of pyranopyrazoles was found to be new and understudied in current literature.

## Experimental section

### Methods and apparatus

All the chemicals and crown ether were commercially purchased and used without further purification. Melting points were recorded using Electronics india 935 Digital Melting Point Apparatus. The ^1^H and ^13^C NMR were recorded using BRUKER 400 MHz and 100 MHz instruments respectively using TMS as reference. The IR spectra were recorded on a SHIMADZU FT-IR-84000 s spectrophotometer.

### General procedure for the synthesis of pyrano[2,3-*c*]pyrazoles using crown ether as a catalyst

To a mixture of aromatic aldehyde **1a–p** (2 mmol), **2** (2 mmol), **3** (2 mmol), **4a/4b** (2 mmol) in water (10 mL) taken in a 50 mL RB flask, crown ether 10 mol% was added. The reaction mixture was subjected to sonication for 10 min at room temperature. The completion of reaction was confirmed using TLC. The solid product was filtered and recrystallized from aqueous ethanol to get the pure products.
